# Near-Infrared Spectroscopy in Bio-Applications

**DOI:** 10.3390/molecules25122948

**Published:** 2020-06-26

**Authors:** Krzysztof B. Beć, Justyna Grabska, Christian W. Huck

**Affiliations:** Institute of Analytical Chemistry and Radiochemistry, Leopold-Franzens University, Innrain 80/82, CCB-Center for Chemistry and Biomedicine, 6020 Innsbruck, Austria; Justyna.Grabska@uibk.ac.at

**Keywords:** NIR, near-infrared spectroscopy, NIRS, biospectroscopy, bioscience, analytical

## Abstract

Near-infrared (NIR) spectroscopy occupies a specific spot across the field of bioscience and related disciplines. Its characteristics and application potential differs from infrared (IR) or Raman spectroscopy. This vibrational spectroscopy technique elucidates molecular information from the examined sample by measuring absorption bands resulting from overtones and combination excitations. Recent decades brought significant progress in the instrumentation (e.g., miniaturized spectrometers) and spectral analysis methods (e.g., spectral image processing and analysis, quantum chemical calculation of NIR spectra), which made notable impact on its applicability. This review aims to present NIR spectroscopy as a matured technique, yet with great potential for further advances in several directions throughout broadly understood bio-applications. Its practical value is critically assessed and compared with competing techniques. Attention is given to link the bio-application potential of NIR spectroscopy with its fundamental characteristics and principal features of NIR spectra.

## 1. Introduction

Near-infrared (NIR; 1000–2500 nm; 10,000–4000 cm^−1^) spectroscopy might be summarized as a vibrational spectroscopy technique [1,2,3] that occupies a somewhat peculiar spot across the field of bioscience. On the one hand, it has earned the status of tool-of-choice in various applications concerning the qualitative and quantitative assessment of bio-related samples, e.g., in medicinal plant analysis or the issues related with the quality control of natural products. On the other hand, in several others areas it competes with better established techniques such as infrared (IR, i.e., mid-infrared, MIR; 4000–400 cm^−1^; 2500–25,000 nm) and Raman spectroscopy. The typical key areas are e.g., bioanalytical research and biomedical diagnosis, in which NIR spectroscopy has typically been shadowed by IR and Raman techniques [4,5,6,7,8], and where it still has room to progress in popularity. As the consequence of several reasons, with some exceptions, the full potential of NIR spectroscopy in bio-applications yet remains to be uncovered. Nonetheless, NIR spectroscopy steadily gains in importance with a number of recent advances accelerating this trend. Recent years have demonstrated that NIR spectroscopy may successfully be used in unique ways and deliver information difficult to obtain by the competing techniques.

The present review aims to provide a critical overview of NIR spectroscopy in a diverse field of bio-applications, and to expose its strengths and limitations in comparative manner. Recent literature lacks comprehensive reviews that would aim to sketch the current state and the future potential of NIR spectroscopy in this area of research. For this reason, it is useful to briefly summarize the essential similarities and differences existing between these competing techniques. Aspects discussed include the physical background and implications of the key advantages of NIR spectroscopy, such as minimal requirement for sample preparation, suitability to interrogate moist samples, potential for accurate quantitative analysis, etc. At the same time, a closer look at the current limitations will not be avoided. Hindering factors are discussed, e.g., inferior chemical specificity of NIR spectroscopy compared to IR or Raman spectroscopy, less straightforward spectra interpretation reducing useful correlations between NIR spectra and the complex molecular composition of a biological sample, or enhancement of selected vibrations (X-H groups) in the spectra [3,9]. Certain features may be deemed either beneficial or disadvantageous, depending on the application; here, deep sample penetration by NIR radiation and the evaluation of the sample in greater volume should be mentioned. This makes NIR spectroscopy relatively better suited for in vivo examinations, a feature particularly important in biomedical applications [10]. Special attention is given to evaluate the recent accomplishments in mitigating the method’s limitations and improving its applicability, and its future development directions.

The discussion is based on a systematic cross-section of a diverse field of bio-applications of NIR spectroscopy based on published literature. The assumed categorization only serves the need to maintain clarity of presentation, as the research activity in the field of NIR spectroscopy often has boundary-crossing character [1]. Investigations into properties of biological samples ranging from cells, through tissues to entire organisms are reviewed. Separately, analyses of body fluids are inspected as these have practical importance in a variety of fields, e.g., biomedical or forensic science. NIR spectroscopy is a recognized tool for analysis of plant-related samples including plant medicines, and therefore, a considerable attention will be given to this area of applications [11]. Worth mentioning is the extremely vibrant clinical application of functional NIR spectroscopy (fNIRS) in medical diagnosis, where it serves the purpose of functional neuroimaging. In addition, NIR imaging spectroscopy is a potent tool capable of providing spatial distribution of chemical composition of the sample. Spectral imaging is extremely useful for studies of biological samples and novel approaches are feasible to monitor in vivo dynamical processes occurring in the sample. Within the current review, it is not possible to exhaustively present the basic principles for the immensely diverse field of bio-applications of NIR imaging techniques. Instead, a brief introduction and selection of relevant examples will be provided with aim to provide basic understanding of the potential that these techniques offer in bioscience. Interested readers seeking a more complete overview of NIR spectral imaging will be pointed to focused literature. For similar reasons, this review does not include any introduction to data-analytical methods, spectra pre-processing and chemometrics. These tools have a critical importance in NIR spectroscopy, and the interested reader is pointed to rich comprehensive literature covering these topics; e.g., fundamental text-books presenting chemometrics [12,13]. Noteworthy is a recent tutorial on NIR spectroscopy by Pasquini [14], discussing instrumentation, experimental and data-analytical tools, as well as imaging techniques.

## 2. Principles of NIR Spectroscopy from the Point of View of Applications in Bioscience

**Figure 1:**
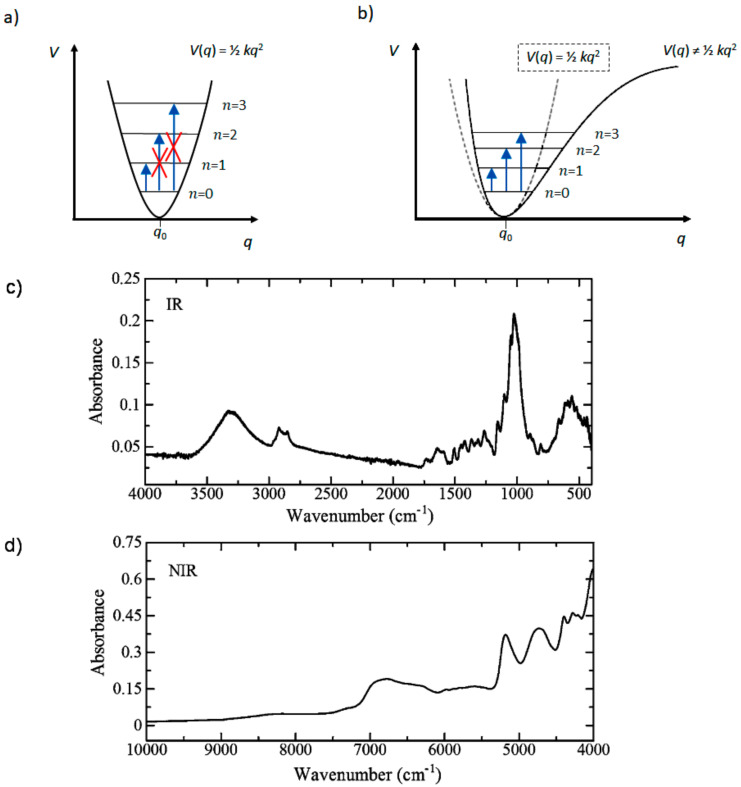
(**a**,**b**) Vibrational potential, vibrational levels and transitions of diatomic (i.e., one-dimensional) oscillator in (**a**) harmonic approximation, and (**b**) its real (anharmonic) nature, (**c**,**d**) comparison of infrared (IR) (**c**) and near-infrared (NIR) (**d**) spectra of the same sample (wood of Douglas fir species). The symbols denote: *V*—the potential energy; *q*—vibrational coordinate; *k*—force constant; *n*—vibrational quantum number. Panels (**c**,**d**) reproduced with permission from Springer Open, Ref. [16].

Together with infrared (IR) and Raman, NIR spectroscopy belongs to the field of molecular vibrational spectroscopy, in which the interaction with electromagnetic radiation probes the vibrational (i.e., internal) degrees of freedom (DOFs) of molecules. Those DOFs correspond to oscillating changes in bond lengths and angles between these bonds, or in other words stretching and deformation vibrations (modes), respectively. The molecular oscillations feature characteristic frequencies ω that correspond to wavelengths of incident electromagnetic radiation, at which absorption phenomenon may occur resulting in an appearance of an absorption band in the spectrum of a molecule. However, one’s attention needs to be directed at the fundamental difference between NIR spectroscopy and the other techniques. The most approximate description of molecular vibration is given by the model of the classical harmonic oscillator for which a characteristic feature is the approximation of the true vibrational potential (i.e., vibrational energy) by a quadratic function (parabola; Figure 1a). The harmonic approximation is an efficient approach for describing the fundamental frequencies (*ω*), i.e., those responsible for the bands appearing in IR or Raman spectra (the wavenumber region of fundamental transitions is commonly given as ca. 4000–400 cm^−1^). Note, in the region below 400 cm^−1^ (far-infrared, FIR) one can observe fundamental torsional and skeletal vibrations [15]. In contrast, NIR region (12,500–4000 cm^−1^) comprises non-fundamental bands, overtones (e.g., first overtones 2*ω*, second overtones 3*ω*, etc.) and combination bands (e.g., binary sum combinations *ω*_a_ + *ω*_a_, ternary sum combinations *ω*_a_ + *ω*_b_ + *ω*_c_, etc.). This cornerstone difference dictates distinct disparity in the applicability of NIR spectroscopy to various problems encountered in the fields of bioscience; a brief explanation of this key difference is presented in Section 2.1.

### 2.1. Merits and Pitfalls of NIR Spectroscopy in Comparison with Competing Techniques

The appearance of non-fundamental bands (e.g., as listed in Table 1) is the result of the anharmonic nature of molecular oscillators (Figure 1b). Anharmonicity of molecular vibrations is critical for understanding the characteristics of NIR spectroscopy. Here, the features essential in the applications throughout the field of bioscience will be emphasized (Table 2). Note, Raman spectroscopy differs by its physical principles, and for clarity only its practical factors will be summarized in this Section. The interested reader is referred to comprehensive book chapters and review articles on Raman spectroscopy in bio-applications, e.g., by Barańska and co-workers [5,6,7,8]. The number of non-fundamental transitions, mostly the combinations, is significantly higher than the fundamental ones. Hence, in NIR spectra the number of individual bands is markedly higher than in IR spectra. Further, NIR bands tend to strongly overlap creating broad absorption profiles (compare Figure 1c,d). The identification of the contributing vibrations is very difficult, and often only approximate NIR band assignments are available [17]; this makes NIR spectroscopy inferior in chemical specificity to IR or Raman techniques. Approximate wavenumber regions, in which NIR absorption bands may appear for biological samples, are summarized in Table 1. Noteworthy, with quantum chemical calculations it is possible to interpret NIR spectra in detail [9]; examples concerning bio-applications are provided in Section 3.7.

The probability of a non-fundamental transition to occur is significantly lower than a fundamental one. Therefore, the absorptivity of organic molecules in NIR region is markedly lower (by a factor of ca. 10^1^–10^2^) than in IR. Consequently, the penetration depth of the incident radiation up to the point of complete absorption at the wavenumbers of most intense bands is distinctly higher in the NIR region (typically few mm) than in IR (typically few μm). Hence, NIR radiation senses a much higher volume of the sample beneath the immediate surface. For this reason, IR and NIR spectroscopy differ remarkably in their typical sampling depth, or in other words, in how the information is collected from distinctively different sample volumes. This fact has a notable consequence, as the typical samples investigated in bioscience are highly inhomogeneous, often micro-structured samples, such as cells and tissues of either plant or animal origin. The consequence of this is well exemplified in medical applications, in which the optimal sample thickness for IR transmission measurements conveniently matches the typical configuration of microtomed tissue specimen used in conventional medical diagnosis. In contrast, no useful NIR signal can be obtained from such specimens. On the other hand, it becomes feasible by using NIR spectroscopy, for example, to probe information from beneath human skin or examine entire organisms such as fish embryo, for which IR spectroscopy could not be applied. Deep tissue sampling is a key advantage for bioanalytical applications of NIR spectroscopy, even though an averaged signal from a larger volume of the sample is acquired. Moreover, as a larger sample volume is suitable for NIR spectroscopic measurement, analysis of bulk materials is more practical (e.g., analysis of natural products) and minor inhomogeneity or surface properties are not detrimental to the accuracy. Strong water absorption forms a limitation for IR studies of biological samples; it may be overcome by using attenuated total reflection (ATR). Here, NIR spectroscopy is relatively better suited for examination of samples with high water content. Measurements in transmission or diffuse reflection mode without sample preparation are more feasible than in IR spectroscopy. Compared with Raman, which is suitable for interrogating moist samples, NIR technique is applicable to specimen with high content of fluorophores; those most typically encountered in biosamples are e.g., chlorophyll or proteins. Consequently, NIR spectroscopy is easily applicable for examination of plants and plant-related materials, as well as protein-rich samples. Finally, greater suitability of the principal features of NIR instrumentation in certain applications should be highlighted. Availability of fiber optic probes suitable for the NIR region makes remote operation or in vivo diagnosis easier. A considerable market of miniaturized NIR spectrometers is established, with new improved devices appearing almost every year. In contrast, such IR sensors are practically limited to ATR, and their application faces difficulties because of the stability of sampling conditions. The selected practical aspects and relevant comments on principles and applicability of NIR spectroscopy in comparison with the other vibrational spectroscopic techniques are summarized in Table 2.

Detailed introduction to the instrumentation falls outside of the scope of present review; however, comprehensive information on this topic is available in recent literature [2,18]. Nonetheless, mentioned should be some design features of NIR spectrometers that make this technique highly attractive for biological and biomedical applications in practice. The miniaturized technology of NIR spectrometers is considerably more affordable that competitive techniques [19]. Unlike for IR or Raman spectroscopy, fiber optic probe technology is readily available for NIR wavelengths. This enables inexpensive NIR instrumentation for remote operation via small and flexible fiber optic probe sensor. Unlike ATR-IR approach, non-contact analysis of various samples with no or minimal preparation is feasible in NIR spectroscopy. Further, if diffuse reflection mode yields unsatisfactorily low signal, the effective optical path may be increased two-fold using transflection mode with reflective element placed behind the sample; however this approach is often limited to in vitro examinations. Because the absorptivity in NIR region varies, being higher in the region of the first overtones and binary combinations and lower in the region of higher order transitions, one can more flexibly select an optical path length or wavenumber range to fit the need of the study. For those reasons, NIR spectroscopy can be more conveniently applied to samples of various state, shape and thickness [18].

## 3. Overview of Applications

### 3.1. Analysis of Body Fluids

#### 3.1.1. Blood

The examination of blood has been one of the earliest applications of NIR spectroscopy in medical diagnosis. Early adoptions include the investigation into oxygenation level of metabolites in blood pioneered by Jobsis in 1977 [20]. The development of NIR spectroscopy for the purpose of blood analysis has accelerated in the nineties with numerous trend-setting studies. The available instrumentation and proposed methods enabled more quantitative and direct measurements. For example, as demonstrated by Ozaki et al. in 1992 [21], NIR absorption provides accurate information for the determination of deoxyhemoglobin concentration in venal blood. As the result of these early advances, NIR spectroscopy is nowadays a well-established tool for the analysis of blood and body fluids. Nonetheless, it remains an active and continuously progressing area of research.

##### Glucose in Blood

Although fairly matured, the analysis of blood glucose by NIR spectroscopy continues to be an intensively studies topic with several recent literature reports reflecting the attention given to this field and the dynamics of the research. Recently, Uwadaira et al. re-evaluated in detail the suitability of the absorption bands in LW-NIR (long wavelength) vs. SW-NIR (short wavelength) for blood glucose examination [22,23]. Typically, the LW-NIR region (ca. 7700–4000 cm^−1^; 1300–2500 nm) is used in blood glucose analysis. LW-NIR consist of the combinations or the first overtones of the OH, CH, and NH modes (Table 1). These bands are sharper than those in SW-NIR, which arise from higher overtones and combinations. However, the absorption index of LW-NIR bands is notably higher, and therefore, only shallow penetration depths is effectively achieved. Often this limitation makes mostly interstitial fluid in the epidermis layer of the skin being measured instead of deeper located tissues and blood vessels. In SW-NIR, however, there appears an absorption gap between visible absorption of hemoglobin and NIR absorption of water, creating an optical window for sensing the deeper layers of the tissue. The studies by Uwadaira et al. [22,23] demonstrated that the correlation between SW-NIR spectra and blood glucose content is sufficient to successfully construct calibration models based on partial least squares (PLS) regression. However, this could reliably be done only for individual subjects. It was observed that instead of directly using the change of blood constituents, a more effective for the development of a successful calibration model was body mass index (BMI). Interestingly, BMI seemingly affects the physical measurement conditions for blood glucose level. However, for certain subjects the individual calibration models were unsuccessful, leaving room for improvement towards robustness of this method. With aim to address this issue, the authors continued their research and proposed a simple approach in which a large data set containing ca. 400 carbohydrate tolerance tests (CTTs) was used [23]. It suits systematic evaluation of every wavelength in NIR spectrum, if a direct correlation between blood glucose level and NIR absorbance can be determined. This approach successfully established NIR wavelengths at which absorption is strongly correlated to the blood glucose level. However, daily fluctuations even for the same person were observed at these wavelengths. As reported, the method is reliable for a 2-h analysis period. Therefore, further advances are needed to develop a more robust and universal prediction model. Further studies from other groups aimed at determining the informative wavelengths in the NIR region for non-invasive blood glucose prediction should be noted, e.g., Yang et al. [24], or Suryakala and Prince who used PLS regression and principal component regression (PCR) models for this purpose [25]. Non-linear regression by means of artificial neural network (ANN) has also been evaluated and compared with PLS regression by Jintao et al. [26]. This demonstrates well the room for improvement that still exists in this seemingly well-established application field of NIR spectroscopy.

Much attention is paid to refining the chemometric analysis in this application. Improvements in accuracy and robustness are proposed, e.g., towards reducing the number of independent variables in calibration models for glucose concentration and correcting the individual physiology-related differences and dynamics of glucose, as reported by Dai et al. [27]. A set of two ANNs combined with particle swarm optimization (PSO), was proposed as a nonlinear calibration approach to this problem. The weight coefficients of the two ANNs, which represented the differences between individual and daily physiological rules, were optimized by PSO. This strategy successfully overcame individual differences and physiological glucose dynamics and thus, enhanced the robustness of predicting glucose concentration in blood.

It is worth to highlight the progressing applicability of NIR spectroscopy as a competing technique in the medical applications typically dominated by other tools. For example, blood-oxygen-level-dependent contrast functional magnetic resonance imaging (BOLD-fMRI) is a favored technique for detection of brain cancer. Nonetheless, this method faces some limitations. In some cases of brain disorder in previous studies, e.g., stroke and brain tumor, BOLD-fMRI diagnosis had been demonstrated to produce incorrect image of activation areas. Sakatani et al. adopted NIR spectroscopy for improving the diagnostic reliability in such cases [28]. The study compared NIR spectroscopy and BOLD-fMRI results of functional brain activation in patients. The characteristics of the cerebral blood oxygenation (CBO) changes corresponding to stroke and brain tumors were monitored by both techniques. Essentially, NIR spectroscopy offered a major improvement and delivered good diagnostic performances in the cases, where BOLD–fMRI performed unsatisfactorily. The study evidenced that application of NIR spectroscopy leads to superior accuracy and reliability in the functional imaging of diseased brains, in the cases where established techniques face limitations [28].

##### Blood Oxygen Level

In spite of this field being one of the earliest adoptions of NIR spectroscopy in medical diagnosis, recent literature indicates that it remains an active area of research with novel concepts being proposed. The specificity and sensitivity of NIR absorption to hemoglobin creates rich opportunities for non-invasive diagnosis. Recently explored directions include NIR imaging techniques for real-time in vivo visualization of the chemical distribution of hemoglobin with differing properties. For instance, Mehnati et al. [29] developed a method of NIR multispectral imaging for optical differentiation of vessels according to hemoglobin concentrations. Successful application of this methodology for locating targeted vessels for mammography was limited, as the discrimination between vessels with various hemoglobin concentrations needs to be further enhanced. Nonetheless, good image contrast was obtained, with promising prospects for early diagnosis and prescreening breast cancer.

Nioka et al. developed an approach based on NIR spectroscopy for breast tumor diagnosis in patients undergoing biopsy [30]. Continuous SW-NIR spectroscopy was employed to measure blood volume and blood hemoglobin concentration. The study aimed to verify whether angiogenesis and hypoxia are meaningful factors for cancer detection. This would be possible, if the correlated parameters, the total hemoglobin content and oxygen saturation, can be used as biomarkers for those clinical conditions. The study revealed, that by monitoring high total hemoglobin and hypoxia scores, the sensitivity and specificity of cancer detection could be maintained at 60.3% and 85.3% levels, respectively. It was concluded that smaller-size tumors are more challenging for detection by NIR spectroscopy, whereas ductal carcinoma in situ (DCIS) can be detected using configurations presented in the study. It was noted that for larger-size tumors, a significantly higher deoxygenation occurs in DCIS than in benign tumors [30].

Interestingly, skeletal muscles can also be analyzed to obtain useful information on the oxygen level in blood. This direction was recently discussed in detail by Chatel et al. [31]. Medical applications do not solely require the analytical tools for determination of blood oxygenation in vivo. A good example is the non-destructive measurement of hemoglobin in blood bags, as reported by Zhang et al. where this was accomplished using multipath-length VIS-NIR spectroscopy [32]. A major difficulty in such application is the complex spectral and optical properties of blood bag material which require sophisticated approaches to yield accurate information on the contained blood.

Noteworthy is the method based on refractometry for the analysis of blood oxygen level. Interestingly, not only blood absorption but also refraction can be used for this purpose. The refractive index of the hemoglobin solution is indicative for its oxygenation. Lazareva et al. [33] accomplished good results through measurements of the refractive index values at 480, 486, 546, 589, 644, 656, 680, 930, 1100, 1300, and 1550 nm wavelengths covering the visible/near-infrared (NIR) region. Laser emission lines and multi-wavelength Abbe refractometer were employed for measuring hemoglobin aqueous solutions of different concentrations. Hemoglobin was obtained from human whole blood. The study reported specific increments of refractive index correlated with hemoglobin concentration from which the Sellmeier coefficients were calculated [33].

##### Blood Stain

Blood stain analysis, in forensics, largely benefits from on-site capable methods. The miniaturized, affordable NIR spectrometers available nowadays, suit this role perfectly. Therefore, major focus is currently given to the adoption of the portable devices for this role. For instance, Pereira et al. [34] developed a noninvasive, nondestructive method based on a hand-held NIR sensor for confirmatory and in situ identification of dry blood stains on different substrates. The samples included human and animal blood stains. Additionally, for simulating potential false positives, stains from different commercial products that may resemble blood stains were used. The study involved a sophisticated suite of data-analytical methods. Pre-processing methods (standard normal variate, SNV; and normalization by range) preceded application of several supervised pattern recognition methods. The highest accuracy for discriminating human blood stain was achieved with the soft independent modeling of class analogy (SIMCA) algorithm, with resulting 100% correct classification for porcelain and glass, 80% for metal, and 90% for ceramic as substrates. Comparative methods involved PLS discriminant analysis (PLS-DA), genetic algorithm-linear discriminant analysis (GA-LDA), and successive projection algorithm-linear discriminant analysis (SPA-LDA). This demonstrated the suitability of on-site NIR spectroscopic detection of human blood stains on various substrates.

##### Hemodialysis

**Figure 2:**
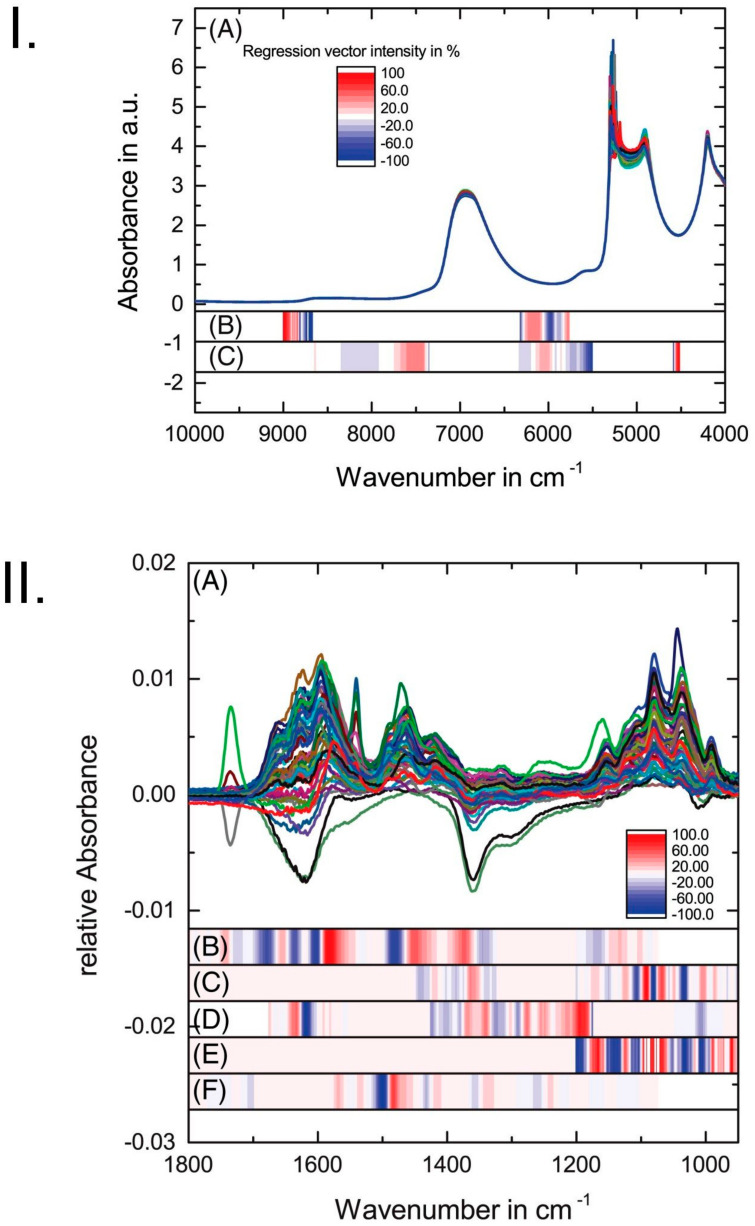
Dissection of the PLS regression vectors developed by Henn et al. [33] for prediction of blood constituents from NIR absorbance (**I.A**) and IR difference (**II.A**) spectra of a 5-component model mixture in artificial dialysate solutions. Relative (to the maximum value) intensity of the regression vector for glucose (**B**) and urea (**C**), lactate (**D**), phosphate (**E**) and creatinine (**F**). Adapted in agreement with CC BY 4.0 license, Ref. [35].

Hemodialysis can successfully be monitored in detail by IR and NIR spectroscopy as reported recently by Henn et al. [35]. The authors evaluated the suitability of both spectral techniques and compared their performances when used in hyphenation with PLS regression. The aim was to analyze quantitatively the blood constituents such as urea, glucose, lactate, phosphate and creatinine. These are important markers for the process of detoxification, particularly in ambulant dialysis treatment. The study aimed to compare IR and NIR techniques to determine the targeted molecules quantitatively in artificial dialysate solutions. These methods were directly assessed in accuracy by means of statistical errors determined in PLS regression analysis based on the same sample set. Noteworthy, the study included a detailed analysis of the wavenumbers meaningful for this purpose in both IR and NIR regions. The authors dissected the structure of the PLS regression coefficients vector developed for quantification of the target analytes in the sample on the basis of IR and NIR spectra (Figure 2) [35]. This detailed analysis unveiled that there are relatively few NIR meaningful wavenumbers in the regression models of glucose and urea concentration in artificial dialysate solutions. Interestingly, these wavenumbers are located in the regions free from strong absorption bands of water (Figure 2I). In order to take into account the variations in the concentration levels during dialysis, a multilevel/multifactor design was employed. The conclusions from those results were that IR spectroscopy is better suited to analyze the blood constituents; urea, glucose, lactate, phosphate and creatinine. This technique employed in a multi-reflection attenuated total reflection (ATR) mode enables a reliable prediction of all five target analytes under investigation. At the same time, NIR spectroscopy was successful only in the determination of urea and glucose. However, NIR spectroscopy provides considerable practical advantages such as easy sampling, or potential use of miniaturized sensors. Nonetheless, for both IR and NIR analyses, the coefficients of determination *R*^2^ in PLS regression of at least 0.86 were achieved, as determined in the test-set validation (TSV) procedure for urea and glucose. The method applied to the analysis of lactate, phosphate and creatinine performed well in the IR region with *R*^2^ ≥ 0.95 using TSV (Table 3). This study indicates that ATR–IR and NIR techniques are readily available for glucose and urea analysis. Yet, there exists room for improvement in the performance levels of NIR spectroscopy applied to determination of lactate, phosphate and creatinine (Table 3).

#### 3.1.2. Serum

**Figure 3:**
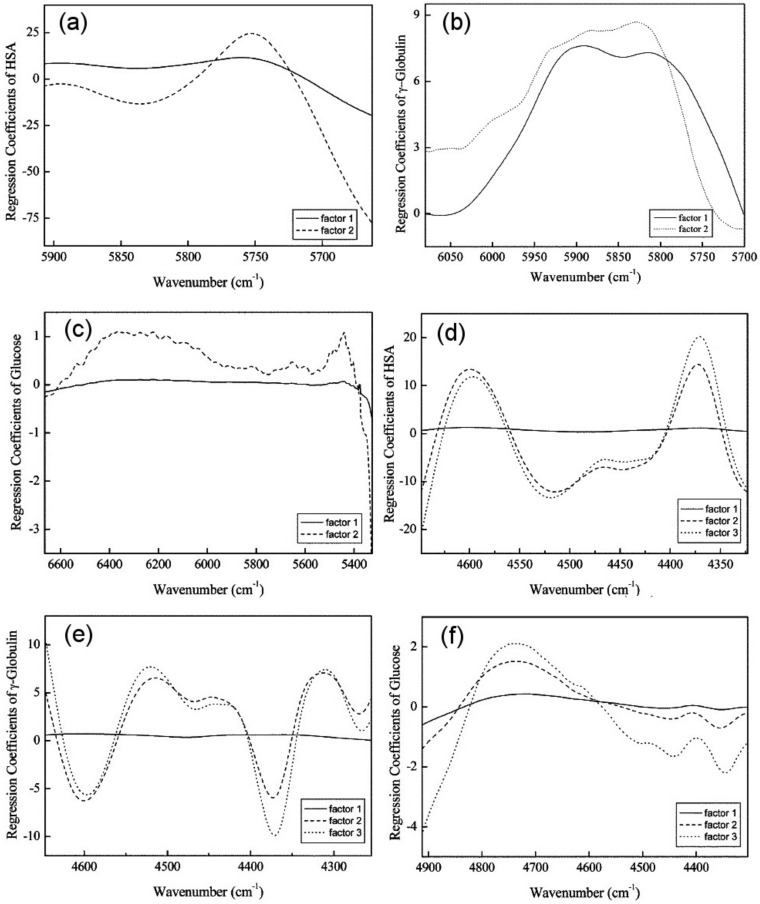
The structure of PLS regression coefficients vectors for simultaneous determination of HSA (**a**,**d**), γ-globulin (**b**,**e**), and glucose (**c**,**f**) concentrations from NIR spectra of model solutions developed by Kasemsumran et al. Ref. [36]. Reproduced with permission from Royal Chemical Society, Ref. [36].

Protein composition and glucose content in serum were frequent objects of NIR spectral studies. For example, Kasemsumran et al. conducted systematic examination of the analytical capability of NIR spectroscopy in the analysis of human serum albumin (HSA), γ-globulin, and glucose for the needs of biomedical purposes [36,37]. The aim of these studies was to evaluate the potential of NIR spectroscopy for simultaneous determination of HSA, γ-globulin, and glucose in matrices of different complexity. Firstly, a model phosphate buffer solution was used and subsequently, control serum solution was included as it better represents a complicated biological fluid. In the model case of phosphate buffer solution, five levels of full factorial design were used to prepare a sample set consisting of 125 samples of three component mixtures, which were examined at 37 °C at various concentration levels. The acquired spectra were analyzed by means of a moving window PLS (MW–PLS) regression algorithm. This approach has been designed for locating the most informative spectral intervals over the measured spectral region; this procedure builds a series of PLS models in a window that iteratively moves over the whole spectral region [38]. It has been reported to be helpful in multicomponent spectral analysis, e.g., in the complex chemical matrix such as serum, as MW-PLS calibration model is very stable against the interference from non-composition-related factors [38]. In the case of the investigation by Kasemsumran et al. [36], this approach unveiled the spectral ranges of 4648–4323, 4647–4255 and 4912–4304 cm^−1^ as the most informative and best correlated with the content of the targeted molecules (Figure 3) [36]. In a subsequently continued study, in which the analysis of HSA, γ-globulin, and glucose was performed in a more complex matrix of control serum solution, an evolutionary, even better performing regression algorithm was evaluated [37]. The applied chemometric method, searching combination moving window PLS (SCMW-PLS) was employed to obtain calibration model against HSA, γ-globulin, and glucose in the control serum IIB (CS IIB) solutions with various concentrations. The obtained results evidence the capacity of NIR spectroscopy supported by SCMW-PLS to simultaneously determine the concentrations of HSA, γ-globulin, and glucose in a complex biological fluid [37].

Recently, a screening method for serum albumin based on NIR spectroscopy was proposed by Yao et al. [39]. That study focused on the wavelength selection for an efficient and rapid NIR analysis of human serum albumin. The number of samples used in the study was 170 divided into two batches, each of which was acquired in different days. The reference analysis was performed in accordance with the routine clinical method using a Hitachi 7060 automatic biochemical analyzer; reference concentrations of human serum albumin ranged from 20.90 to 54.60 (g L^−1^), with the mean value and standard deviation of 39.30 and 5.81 (g L^−1^), respectively. With aim to improve the prediction performance of the analyte in complex matrix, a wide selection of chemometrics was employed. These methods included equidistant combination PLS (EC-PLS), its derivation in the form of repetition rate priority combination PLS (RRPC-PLS), and competitive adaptive reweighted sampling combined PLS (CARS-PLS) as wavelength selection methods. The stability of the method’s parameters was evaluated through modeling of human serum albumin concentration on the basis of a variety of calibration and prediction sets. To ensure an objective evaluation of the performances, validation of the models was based on test-set sample group previously excluded from calibrations. The best performing models included N = 32, 36 and 24 wavelengths, for CARS-PLS, EC-PLS, and RRPC-PLS methods, respectively. However, good prediction power was also achieved by a simpler RRPC-PLS model with just 15 wavelengths selected. Comparing the performances of the more complex and simpler RRPC-PLS models, the root-mean-square errors (RMSE) and correlation coefficients for validation were 0.505 g L^−1^ and 0.997 for the optimal model (N = 24), and 0.530 g L^−1^ and 0.997 for the simpler model (N = 15). The validation indicated better performance of RRPC-PLS compared with CARS-PLS and EC-PLS, in regard to the model complexity and prediction accuracy, with predicted values nearly matching the reference values. Therefore, the conclusion was that RRPC-PLS method is a notable improvement over EC-PLS in application to NIR spectral determination of human serum albumin.

#### 3.1.3. Saliva

Instead of blood or serum, saliva is an alternative source of diagnostic information useful in examining various conditions, e.g., cancer, diabetes, or oral leukoplakia. Compared with blood or serum, saliva is a less complicated matrix with less variable chemical composition. Yet, it contains proteins, nucleic acids, mucins, amino acids, enzymes, and primary metabolites, which are highly informative biomarkers for various physiological conditions of the body. The ease and convenient acquisition of a sample from patients, makes it particularly suited for application of rapid screening method by means of spectroscopy. For those reasons, saliva carries a notable diagnostic potential useful and very practical for biomedical applications of NIR spectroscopy. Consequently, the technique was adopted relatively early for salivary biomarker profiling [18]. Oral cancer cells appear in saliva at early stages of cancer, while oral epithelial cells are transmitted into it continuously during the cancer development. This makes saliva analysis sensitive subject for analysis in oral cancer detection at early stages. For instance, Murayama et al. proposed a diagnostic method based on NIR spectroscopy for detection of oral cancer from one drop of saliva without any specific diagnosis marker [40]. In that study, the NIR spectra of one drop of saliva were measured using a capillary tube method. Principal component analysis (PCA) with the second and third factors calculated with the second derivative NIR spectra clearly discriminated between the two groups [40].

It is noteworthy that the absorption profile of saliva in the NIR region is highly essential for in vivo studies of oral cavity. Methods involving saliva are also in the scope of functional NIR spectroscopy (Section 3.8) studies, e.g., refs [41,42]. Nonetheless, it may be noted that in the field of analysis of body fluids, IR and Raman techniques found great usefulness [43], while the full potential of NIR spectroscopy has not yet been entirely uncovered.

### 3.2. Cell-Related Studies

IR and Raman spectroscopy are matured tools for carcinoma diagnosis. In this field, NIR spectroscopy is advancing and feasible methods are becoming well established. Early developments of NIR spectroscopy at that direction include approaches to detect prostate cancer cells. NIR calibration models for the analysis of glucose, lactate, glutamine, and ammonia as the prostate cancer markers were established by Rhiel et al. [44]. For the calibration, an adaptive procedure was developed with aim to selectively remove interfering metabolism-induced covariance between glucose, lactate, glutamine, and ammonia that arose in the cultivar of PC3-human prostate cancer cells. PLS regression models were generated from single-beam NIR spectra measured in the region of 4800 and 4200 cm^−1^. In the first attempt, the calibration models were developed on the basis of full spectral range; however, in the next steps an optimization of spectral windows was carried out and used for fine-tuned calibrations. This enabled lowering the standard error of prediction (SEP) to 0.82, 0.94, 0.55 and 0.76 mM, respectively for glucose, lactate, glutamine and ammonia. Successful validation of NIR spectroscopy for off-line determination of the concertation levels of nutrients and metabolites in a serum-based cell culture medium formed an important step. Further, benefits from performing cell separation prior the spectral analysis became apparent as well. Such treatment was developed for on-line monitoring of human prostate cancer cells [45]. A polypropylene filter was used for retaining the cells upon centrifugal filtration in that case.

**Figure 4:**
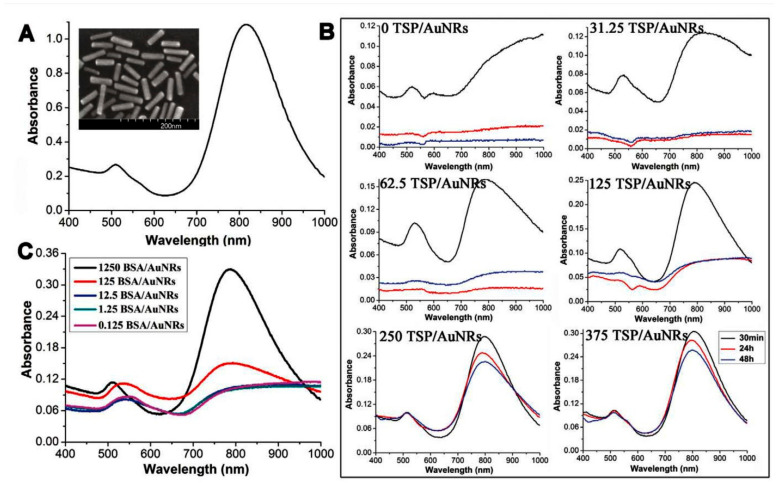
UV-vis-NIR spectrum of AuNRs suspension in water (**A**). Spectra of gold nanorods (AuNRs) dispersed in serum containing media (SCM) with 0–30% of fetal bovine serum (FBS) and different incubation times (**B**). Spectra of bovine serum albumin (BSA)/AuNRs samples (**C**). Reproduced in agreement with CC BY 4.0 license from Ref. [46].

Applicability of electronic absorption spectroscopy extending to NIR region to characterize breast cancer cells was studied by Zhang et al. [46]. The investigation focused on the behavior of gold nanorods (AuNRs) in metastatic breast cancer cells. Of particular note, this has been done in an extended ultraviolet-visible-NIR (UV-Vis-NIR) region (25,000–10,000 cm^–1^; 400–1000 nm). It is an interesting example of how electronic absorption bands, typically studied in UV-Vis region (400–800 nm), that extend to NIR region can be used for practical bio-applications (Figure 4). UV-Vis-NIR absorption spectra of AuNRs in the living cells were used together with the information gained from other techniques (inductively coupled plasma mass spectrometry, ICP-MS; transmission electron microscopy, TEM) to monitor the properties of AuNRs in a highly metastatic tumor cell line. It was observed that the characteristic surface plasmon resonance (SPR) peaks of AuNRs can be detected with living cells that have taken up the nanorods. Further, the peak area of transverse SPR band was determined to be proportionally related to the amount of AuNRs in the cells, giving the developed method quantitative character of analysis. The established easy-to-use UV-vis-NIR absorption spectroscopic method can be used to monitor the behavior of AuNR. Zhang et al. have demonstrated how this capacity can be successfully applied to monitor the appearance of metastatic breast cancer cells [46].

An interesting problem of the appearance of biological cells in milk was examined as a factor potentially influencing the performance of NIR spectroscopy in food analytical applications. Tsenkova et al. investigated the effect of high somatic cell count (SCC) index on the accuracy of NIR spectroscopic determination of fat, protein and lactose content in non-homogenized cow milk [47]. In that study, transmittance spectra of 258 milk samples were analyzed in SW-NIR region (14,285–9090 cm^−1^; 700–1100 nm). The most accurate calibrations, as evaluated through analyzing SEP values and the correlation coefficient, were obtained for the samples with low SCC index. In contrast, the accuracy decreased notably in the scenario, where calibration models constructed on the basis of low SCC milk were subsequently used to predict the target analytes in samples with high SCC, and *vice versa*. Therefore, high cell content influences the accuracy of determination of fat, protein and lactose content in milk. These observations demonstrated the influence of cell content on the robustness of analysis of the chemical composition in milk by NIR spectroscopy; a factor that needs to be taken into account in similar applications.

### 3.3. Analysis of Tissue

Vibrational spectroscopy, in particular imaging instrumentation, is a remarkably powerful tool for examination of tissues. Noteworthy are the applications in medical diagnosis, where IR and Raman spectroscopy are nowadays matured techniques, with prime importance for carcinoma diagnosis. Much alike the latter, a major attention in NIR biospectroscopy has been given to tissue analysis [18]. From the point-of-view of tissue analysis in biomedical sciences, NIR spectroscopy is conveniently partitioned according to short-wave (SW; 13,333–9090 cm^−1^; 750–1100 nm) and long-wave (LW; 9090–4000 cm^−1^; 1100–2500 nm) NIR wavelength intervals. At short NIR wavelengths, the absorption of hem proteins (hemoglobin, myoglobin, and oxy-derivatives) and cytochrome of the tissue dominate the spectra and provide information concerning tissue blood flow, oxygen saturation and consumption, and the redox status of the enzymes. In the LW-NIR region, the observed absorptions are more complex and indicative for different biomolecules that may appear in tissues (Table 1). Valuable information concerning the chemical composition of the tissue with its main components of lipids, proteins, carbohydrates, and water can be gathered from LW-NIR region.

Tissues are micro-structured samples with topological distribution of features. Therefore, NIR spectral imaging carries particular potential for research and analysis aimed at this direction. The major attention in spectral imaging investigations dealing with human tissues were given to their ability for early detection of cancer. IR imaging focuses on fundamental bands of proteins/peptides, DNA, and lipids [48,49,50,51] as the cancer-related biomarkers. In contrast, in the case of NIR absorption of tumor tissues one can observe significantly higher absorption of the total hemoglobin and water, decreased signal related to lipids and tissue hemoglobin oxygen saturation, and higher scattering coefficient values than normal tissue [52]. A high total tissue hemoglobin concentration reflects angiogenesis and elevated tissue blood volume. Decreased level of tissue hemoglobin oxygenation saturation should be connected with tissue hypoxia driven by metabolically active tumor cells. High water content indicates edema and increased cellularity. Decreased lipid content correlates with displacement of parenchymal adipose. Finally, the differences in scattering results from tumors being composed from on average smaller scattering particles, possibly because of increased epithelial and collagen content in comparison to surrounding normal tissue [52]. NIR absorption provides quantitative and functional information about the chemical components of the tissue. NIR wavelengths at which most relevant chemical information on cancerous tissue is available are summarized in Table 4. On the other hand, scattering profile reflects microstructural features and cellular composition of the tissue. These pieces of information combined, yield exhaustive spectroscopic chemical and biological fingerprint of the tissue useful for clinical decision-making in cancer diagnosis [18].

**Figure 5:**
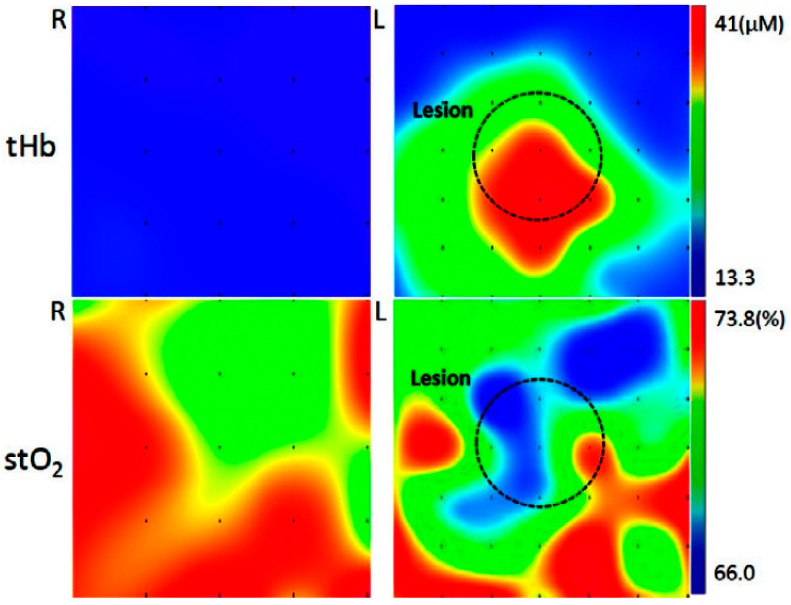
NIR image presenting the distribution of total hemoglobin (tHb) and tissue oxygen saturation (stO2) concentrations on both left and right breast acquired from a 56-year-old subject. Reproduced in compliance with CC BY license from Ref. [53].

NIR spectroscopy, for instance, delivers quantitative chemical information from breast tissue based on oxy-hemoglobin and deoxyhemoglobin, water, and lipids [52]. From these parameters, total hemoglobin concentration and tissue hemoglobin oxygen saturation were determined and provided information on tumor angiogenesis and hyper-metabolism. As an illustrative example, distribution of total hemoglobin (tHb) and tissue oxygen saturation (stO_2_) levels in breast tissue can be monitored non-invasively in NIR spectral images (Figure 5) [53]. The oxygenation indexes (total hemoglobin, deoxy- and oxyhemoglobin, stO2) determined with the use of NIR spectroscopy have been extensively used in exercise (clinical) physiology to answer fundamental research questions concerning muscle tissue oxygenation responses during exercise in humans [54,55]. In addition, expert groups in this area have used NIRS (in conjunction with a tracer) to directly assess absolute and relative values of skeletal muscle perfusion in healthy and patients with chronic diseases [56,57,58,59,60].

The capabilities of NIR spectroscopy, e.g., non-invasive deep tissue sampling, make it potent tool particularly useful in the case of breast cancer. Therefore, considerable attention has been given to this application in the literature. A comprehensive review article discussing in detail early diagnosis and monitoring of breast cancer by NIR imaging is available from Sari et al. [61]. NIR spectroscopy as a diagnostic tool for cancer has been employed for various other cases, e.g., carcinoma of colorectal tissues [62], pancreas [63], or skin [64]. On the other hand, several other relevant chemical features of tissues may be determined by NIR spectroscopy. NIR studies on human tissues include cervix [65], brain [66], skin [67], prostate [68], lung [69], head and neck [70], pancreas [71], and colorectal tissues [63], in which the absorption bands characteristic for one or more of the above mentioned biomarkers.

### 3.4. Analysis of Medicinal Plants and Phytopharmaceutical Applications

The therapeutic and medicinal properties of herbs and plants are most frequently connected with existence of individual bio-active compounds. Hence, their content affects the practical value and general usefulness of any given natural product. The chemical composition and the concentration levels of these bio-active compounds can successfully be analyzed by NIR spectroscopy. One can notice a worldwide increasing trend in using natural drugs derived from medicinal plants [74]. High demand to ensure quality of the natural medicine calls for analytical techniques capable of high-throughput, rapid, non-invasive, simultaneous in situ analysis of chemical and physical parameters of fresh plants, and intermediate or final products. Quality control plays a critical role for medicines derived from plants, as the chemical composition of natural drugs is prone to a much greater variation than conventional pharmaceutical products. Methods based on portable, miniaturized NIR instrumentation are greatly favored in this role, as can be concluded from recent literature [74]. Portable instrumentation enables direct quality assessment and optimization of the cultivation conditions, greatly enhancing the content of the active chemical in the final product, e.g., by selecting the harvest time. However, novel handheld NIR devices differ in the design principles and their applicability and performance profiles of handheld remain continuously investigated in various scenarios [19].

**Figure 6:**
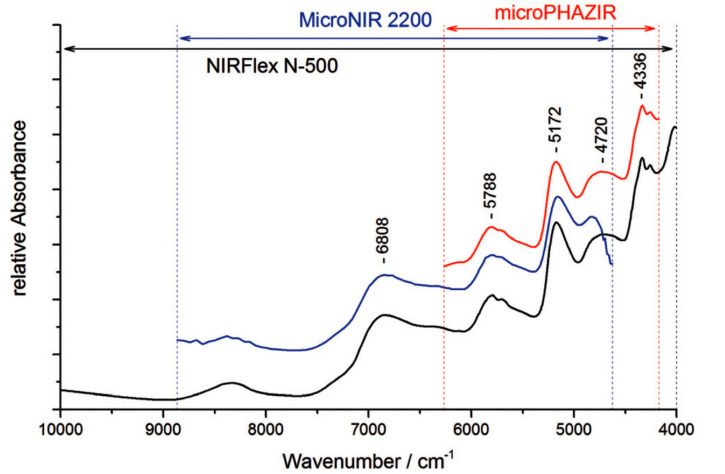
NIR spectra of *Rosmarini folium* samples measured on benchtop (NIRFlex N-500) and handheld (microPHAZIR and MicroNIR 2200) spectrometers. Reproduced with permission from Royal Society of Chemistry, Ref. [75].

**Figure 7:**
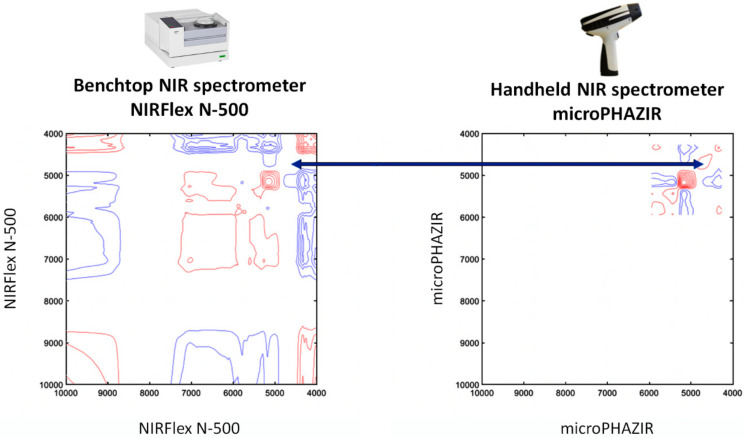
A visual assessment and evaluation of the chemical sensitivity profiles of NIR spectrometers (reference benchtop vs. handheld) by two-dimensional correlation spectroscopy (2D-COS).

For example, characterization of the performance of portable NIR spectrometers in analyzing medicinal plant extracts formed one of the main goals of the study by Kirchler et al. [75]. In their systematic examination, the authors described the capability of miniaturized NIR spectroscopy supported with various tools in determining the antioxidative potential and related properties of natural drugs. The study dissected performances of one benchtop (NIRFlex N-500 FT-NIR, Büchi, Flawil, Switzerland) and two different types of miniaturized NIR spectrometers (MicroNIR 2200, VIAVI Solutions, Milpitas, USA; and microPHAZIR, Thermo Fisher Scientific, Waltham, USA) in the determination of the rosmarinic acid (RA) content of dried and powdered *Rosmarinus officinalis*, folium (i.e., *Rosmarini folium*). Noteworthy, the underlying technology and, thus, the operational parameters (e.g., spectral range, resolution, etc.) and the resulting analytical performance of the various miniaturized NIR spectrometers differ. Therefore, it is an active field of research in NIR spectroscopy to perform systematic evaluation studies of the applicability of certain instruments to various analytical problems. An interested reader is pointed to a focused literature covering the details of the technological aspects and issues related to the performance levels of these instruments [19,76]. The performance profiles were assessed with a number of different data-analytical methods. NIR spectra measured with three spectrometers (Figure 6) were calibrated through PLS regression models against the reference measurements by high performance liquid chromatography (HPLC) (Table 5). Prediction accuracy as determined by cross validation (CV) revealed that the benchtop spectrometer achieved the best result with a *R*^2^ value of 0.91 and a RPD of 3.27. A miniaturized NIR spectrometer, the MicroNIR 2200 achieved satisfying prediction performance with *R*^2^ of 0.84 and a RPD of 2.46. The analysis performed using the handheld microPHAZIR, with a *R*^2^ of 0.73 and a RPD of 1.88, was less accurate and demonstrated room for improvement. In addition to inspecting the PLS regression models, the spectrometers were further evaluated in their sensitivity at different wavelengths by two-dimensional correlation spectroscopy (2D-COS) analysis (Figure 7). The relative differences between the sensitivity of the spectrometers were visualized by 2D hetero-correlation plots. This step allowed identification of discrepancies between the microPHAZIR and the MicroNIR 2200 compared to the benchtop instrument. To gain better understanding of the factors which determine the constructed PLS regression vectors, quantum chemical calculation of the NIR spectrum of RA was carried out. This approach yielded independent information on the NIR absorption features of the target molecule (RA) and enabled interpretation of the main influences in the regression coefficients plots. Furthermore information on applications of quantum mechanics to predict NIR spectra is given in Section 3.7 and in a recent review article [9]. The study by Kirchler et al. demonstrated that the differences between performance profiles of different NIR spectrometers may be better understood with application of different data-analytical tools [75].

**Figure 8:**
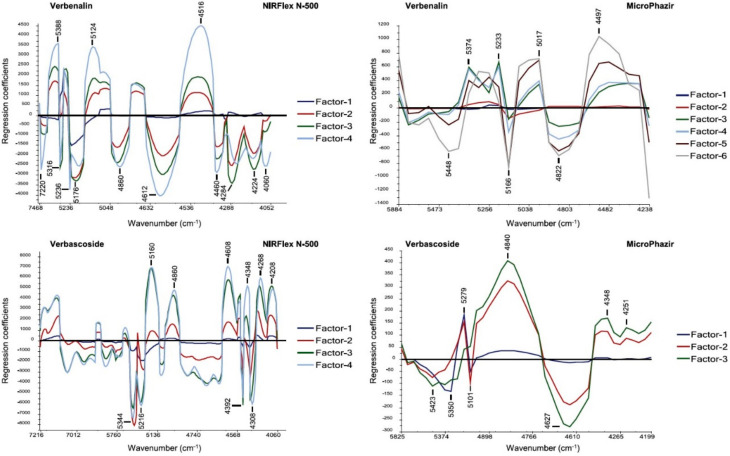
PLS regression coefficients plots for the best performing calibration models for verbenalin and verbascoside content in *Verbena officinalis* samples. The models were constructed for NIR spectra measured on NIRFlex N-500 (benchtop) and microPhazir (miniaturized) spectrometers. Reproduced with permission from Elsevier, Ref. [77].

Optimization of the production parameters of natural drugs is a critical factor in the industry and attracts high attention. As a good example of how laboratory independent NIR spectroscopy can be applied with such benefits, Pezzei et al. compared the suitability of benchtop and portable NIR spectrometers for predicting the optimum harvest time of *Verbena officinalis* [77]. In that study, NIR analyses were performed non-invasively on the fresh plant material based on the quantification of the key secondary metabolite ingredients, verbenalin and verbascoside. NIR spectroscopic measurements were performed using a conventional NIR benchtop device as well as a laboratory independent portable NIR spectrometer, while HPLC served as the reference method. For both NIR instruments, calibrations by PLS regression were established performing cross validations (CV) and test-set validations (TSV). It was concluded that the handheld NIR spectrometer performed comparably with the benchtop device in the determination of verbascoside content. However, calibration for verbenalin content was found to be mildly less accurate. Distinct differences were observed between the PLS regression coefficient vectors constructed on the basis of the spectral data obtained with benchtop and miniaturized NIR spectrometer (Figure 8). This feature likely reflects the operation a characteristics of a portable device, which compared with the reference benchtop, is not only limited in the spectral range, but also in its sensitivity towards absorption features correlated with the analyzed content (Figure 8). This study, therefore, demonstrated that the performance profile of a miniaturized sensor is dependent on the spectral features of the target analyte. Nonetheless, direct measurements on fresh plant material by handheld NIR spectrometer yielded reliable data for quantification of pharmaceutically relevant chemicals while also enabling to determine the optimal time for the harvest.

Delueg et al. developed on-line monitoring strategy for the extraction process of Rosmarini folium based on a combination of wet chemical assays, ultra-HPLC with UV detector (UHPLC-UV) analysis, and NIR spectroscopic analysis method [78]. The combination of the measured NIR spectra and the analytical reference values (total hydroxycinnamic derivatives, total phenolic content and RA content) enabled successful construction of a PLS regression model with coefficients of determination (*R*^2^) of 0.96 obtained by test-set validation. The method was capable of rapid, non-invasive analysis, for on- or in-line process control suitable for application in the sector of natural drugs industry.

The capability of analyzing living plants brings essential benefits to several other fields of practical applications. Natural products often have complex and variable chemical composition. Thus, determination of their quality, origin, detection of adulteration and authenticity check have particular importance. In this area, NIR spectroscopy has become often used, e.g., for quality assessment and authentication of traditional Chinese medicines (TCMs). As an example, Huck-Pezzei et al. could successfully discern pharmaceutical formulations produced from either *Hypericum perforatum* or *H. hirsutum* that originate from China [79]. The analysis done by NIR spectroscopy differentiated plant species, varieties and cultivars, and plants grown in different locations and under different growth conditions.

**Figure 9:**
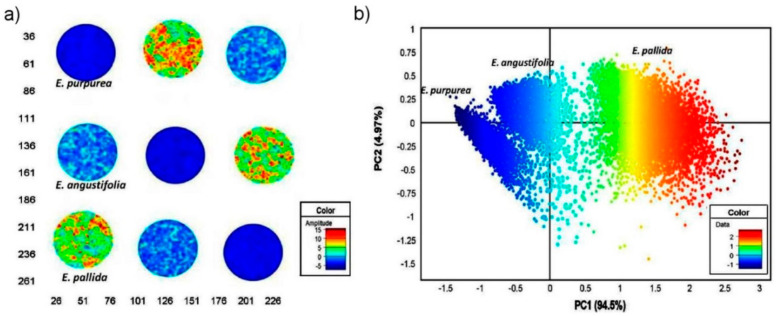
The analysis of NIR images of medicinal plants. PCA score image (t1) of *Echinacea sp*. leaf powders based on color amplitudes (**a**). The corresponding score plot (PC1 vs. PC3) shows minimal separation of the pixel clusters (**b**). (EAL—*E. angustifolia* leaf, EPL—*E. purpurea* leaf, EPaL—*E. pallida* leaf). Reproduced in compliance with CC BY license from Ref. [80].

The potential of NIR spectral imaging for analysis of plant tissues. As they are micro-structured samples, the ability to simultaneously provide chemical and topological information finds a particular usefulness here. As an example, NIR spectral imaging is powerful asset in quality control of natural drugs. This potential has been demonstrated by Sandasi et al. by their method developed for authentication of *Echinacea* based medicines appearing on the pharmaceutical market [80]. *Echinacea* species are often included in various formulations to treat upper respiratory tract infections. The study involved three species, *E. angustifolia*, *E. pallida* and *E. purpurea*, acquired from local market in South Africa. By employing NIR hyperspectral imaging operating in the range 10,870–3978 cm^−1^ (920–2514 nm), with aid of PCA it was possible to clearly discriminate between the three *Echinacea* species from the leaf and root material (Figure 9). The method accurately predicted the raw material content in several commercially available products and identified products that did not contain crude *Echinacea* material as well.

Analysis of chemical compositions and various other properties of plants is often the main aim of various applications in agro-food sector [81]. The potential of NIR hyperspectral imaging is increasing with new approaches to process and analyze spectral images. For example, discrimination of *Chrysanthemum* varieties using NIR imaging was demonstrated by Wu et al. [82]. In that case, deep convolutional neural network (DCNN) was applied to elucidate information from an extremely large volume of data, the spectral images acquired from 11,038 plant samples.

Wood forms a particular type of plant material, the analysis of which by NIR spectroscopy has become well-established. The technique’s capacity to assess multi-constituent chemical, physical, mechanical and anatomical properties of wood beneath just the immediate surface of the sample, in non-invasive manner while retaining the characteristic cellular structure of sample is valued in wood science and technology. A comprehensive review focused on NIR spectroscopy for wood analysis is available from Tsuchikawa and Kobori [16].

### 3.5. Entire Organisms

**Figure 10:**
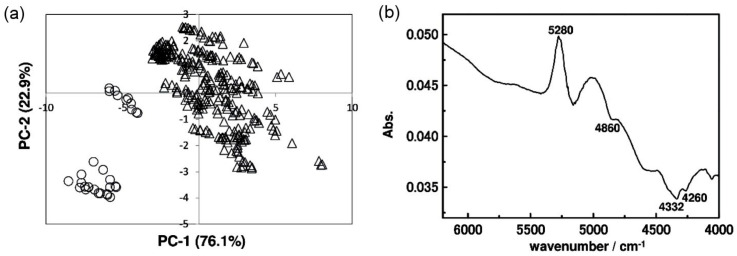
PCA scores plot of NIR spectra of yolk measured over the development time of *Oryzias latipes* embryo (**a**). Δ indicates the data collected from the first to the tenth day and ⚪ denotes data collected at the day before hatching. Loadings plot of PC-1 (**b**). Reproduced from Ref. [83] in agreement with CC BY 4.0 license.

As explained in Section 2, the physical principles underlying NIR spectroscopy make this technique relatively more suitable for deep sample sensing and interrogation of sample in-volume. These features give NIR spectroscopy the capacity for examining entire biological organisms. A good example serves here the recent study by Ishigaki et al. [83]. Therein, the properties of fish embryo have been comprehensively investigated in vivo at the molecular level. The development of fertilized eggs of Japanese medaka fish, *Oryzias latipes*, was monitored by NIR conventional spectroscopy and imaging techniques. The 6200–4000 cm^−1^ region of the NIR spectrum contains useful information on the inner components of the egg, such as proteins, lipids and water. Furthermore over the embryo growth time, oil droplets and yolk undergo changes in their chemical structure, which is accessible for NIR spectroscopy (Figure 10).

Through analyzing NIR spectra and imaging data, one can non-invasively follow the chemical footprint corresponding to metabolic changes occurring in a developing embryo. Insights into subtle features of the chemical environment characteristic to the biological structures of an egg were obtained as well. For example, the study suggested that oil droplets contain relatively more strongly hydrogen-bonded water, and the water environment typical for yolk seem to differ from those found in the other structures. Furthermore, secondary structure of proteins could have been assessed through the characteristic bands at 5756 and 4530 cm^–1^; e.g., the appearance of membrane structures was proposed at certain locations within the egg.

### 3.6. Investigations into Structure, Properties, and Interactions of Biomolecules

NIR spectroscopy is a powerful method for investigation of the molecular structure, properties and interactions, as described by Czarnecki et al. [3]. For the present review, the potential to examine various properties of biomolecules should be stressed. Many of those are complex molecules that often interact with chemical neighborhood, e.g., water as the native environment. For these reasons, the NIR spectral fingerprint of biomolecules is rather difficult for a direct interpretation. Nonetheless, behavior of biomolecules have been successfully studied using sophisticated methods for spectra analysis. For instance, the perturbation-correlation moving-window two-dimensional correlation analysis (PCMW2D) method has shown temperature-dependent structural changes in hydrogen bonds formed by microcrystalline cellulose (MCC) [84]. Additionally, PCMW2D analysis allowed the proposal of band assignments for the OH stretching first overtone region of MCC, and to connect NIR spectral features with hydrogen bonding of different strength. The study continued into an investigation of water adsorption on MCC [85]. PCMW2D and PCA were used to elucidate structural information from NIR spectra of MCC samples with moisture content in 0.2–13.4 wt% range. It was possible to identify strongly overlapping OH stretching bands, and distinguish contributions from MCC and water. The investigation suggested that upon increasing the extent of water adsorption on MCC, a decrease in the free and weakly hydrogen-bonded OH groups, and an increase in the strong hydrogen-bonded OH groups of MCC occur. These observations suggested that inter- and intrachain hydrogen-bonds of MCC are formed through adsorption of monomeric water. Furthermore, ca. 3–7 wt% of adsorbed water seemed to stabilize the hydrogen-bond network formed at MCC–water surface [85].

Significant contributions into protein research have been made with use of NIR spectroscopy; e.g., insights into protein secondary structure [86]. Characteristics of this technique make it highly useful for exploring the hydration process of proteins. The perturbations in the hydration of a protein, as well as its secondary structure can simultaneously be obtained with NIR spectroscopy. Conversely, IR and Raman spectroscopies can hardly investigate these properties at the same time. The capacity of NIR spectroscopy to examine the structure and interactions of proteins in aqueous environment has been recognized relatively early [87,88]. Several methods for analyzing NIR spectra and elucidating information on proteins in aqueous solution were compared by Murayama et al. [87]. The study evaluated the usefulness of conventional spectral analysis methods, chemometrics and 2D-COS analysis. The basis for this evaluation was formed by NIR spectra of human serum albumin (HSA) in 0.5–5.0 wt% concentration range in aqueous solution. Importantly, conventional methods of spectra pretreatment and analysis (e.g., second-derivative, difference spectra), were deemed indispensable for elucidating information on protein highly diluted in water. These approaches were helpful to mitigate the spectral manifestations resulting from complex and not fully understood structure of water. The difference-spectra suggested that the observed gradual concentration-dependent changes in the broad feature in the 7100–6500 cm^−1^ are related to various species of water existing in the sample. Furthermore, the ability of 2D-COS analysis to visualize correlations between individual bands and elucidate sequences of spectral changes was essential in that case.

The exploration of the properties of proteins by NIR spectroscopy was conducted by Yuan et al. who compared different methods for treating NIR spectra of bovine serum albumin (BSA) [88]. However, in that case the temperature variation (45–85 °C) acted as the source of perturbation, while concentration of protein (5.0 wt%) and the pH of the sample (6.8) remained constant. The suite of data-analytical methods was extended by the addition of chemometric algorithm of evolving factor analysis (EFA). That study confirmed the previous conclusions about the usefulness and significance of combined use of various approaches for interpreting NIR spectra. In particular, the difference spectra were critical for unveiling the variations in protein hydration that occurs in the temperature range of 61–65 °C. This finding was possible by analyzing the temperature profile through three-factor EFA that highlighted the 7400–6400 cm^−1^ region as meaningful for the structural change of protein. The examination of NIR spectra by EFA, PCA and 2D-COS led to the conclusion that the change in BSA hydration occurs only after the protein structural variation. It was suggested, that the modification of the hydration structure is triggered by the structural modifications in the protein molecule itself. Interestingly, the NIR spectral manifestations of the structural changes of protein and its hydration shell were different in the case of HSA (concentration variation) [87] and BSA (temperature variation) [88].

NIR spectral bands of proteins carry essential information that enables monitoring complex biological processes in vivo, such as embryonic development [89,90]. In particular, NIR bands are sensitive to hydrogen bonding and thus, to the properties of such molecules in their native aqueous environment as well [3]. Nonetheless, this capability is somewhat limited as the interpretability of NIR spectra of complex molecules such as proteins and other biomolecules remains a challenge, even more so in strongly interacting chemical environments (e.g., water). Often, assignments of only selected NIR band are available, e.g., intense and fairly isolated OH stretching bands. Recent advances in quantum chemical calculation of NIR spectra of biomolecules should be highlighted [9,91,92,93,94], which bring significant progress in the interpretability of their NIR absorption, as well as the insight into how it is influenced by intermolecular interactions. These approaches are essential in improving speculative nature of NIR band assignments (Section 3.7).

### 3.7. NIR Studies Supported by Quantum Chemical Spectra Calculations

NIR spectroscopy has typically been limited by the complexity of the spectra limiting the method’s chemical specificity. This factor is further enhanced in the case of complex biological samples. Even isolated biomolecules have complicated NIR spectra and it is difficult to assign their bands in detail. Recently, progress in applicability of anharmonic quantum chemical calculations to theoretically predict NIR spectra has been reported [9]. This yields significantly improved interpretation of the complex NIR spectra, including biomolecules. In addition, NIR spectra of several biomolecules were successfully reproduced with these methods and their absorption bands were comprehensively explained [9,91,92,93,94]. This approach was also helpful in interpreting the meaningful wavenumbers in PLS regression models of bio-active compounds in plant medicines. The potential stemming from spectra calculation may essentially enhance the inherently inferior chemical specificity, which is one of the few properties of NIR spectroscopy at which it exemplifies a great room for improvements in comparison with IR or Raman spectroscopy. Selected examples relevant to the reviewed topic are presented in the following sub-sections.

#### 3.7.1. Short-, Medium-, and Long-chain Fatty Acids

**Figure 11:**
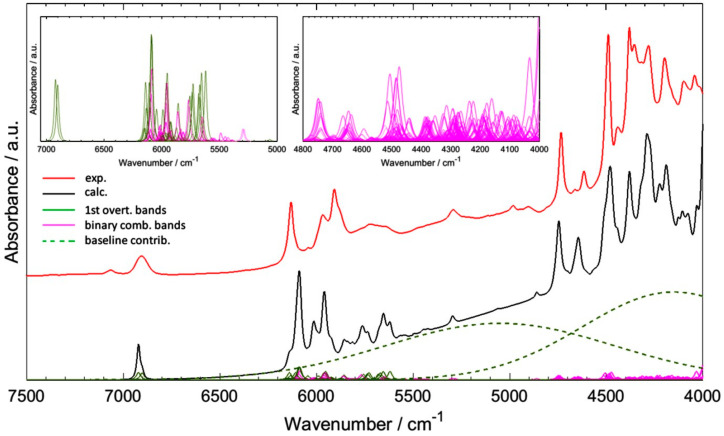
Insight into origins of the NIR spectrum available from quantum chemical calculation on the example of a short chain fatty acid (vinyl acetic acid). Note, all bands are presented in common intensity scale (two inside panels present scaled-up intensity). This demonstrates well the convoluted nature of NIR spectra, which results from strongly overlapping numerous weak bands. Reproduced with permission from Ref. [91]. Copyright (2017) American Chemical Society.

Fatty acids are often the major chemical constituent in a biological sample. Even when not considered as targeted molecules, due to their abundance, the NIR bands from fatty acids may obscure the target signal, e.g., from proteins [89]. Hence, understanding of the NIR spectra of fatty acids is helpful in such cases. Recently, detailed band assignments for NIR spectra of short—(propionic acid, butyric acid, acrylic acid, crotonic acid and vinylacetic acid) [91], medium- (hexanoic and sorbic acid) [92], and long-chain (arachidic acid, palmitic acid, stearic acid, linoleic acid, linolenic acid and oleic acid) fatty acids [93] has become available through quantum chemical calculations. This unveiled characteristic features of fatty acids, contrasting saturated and unsaturated compounds, as well as spectral features depending on the localization of the C=C moiety in unsaturated fatty acids. The previous inability to present a complete cross-section of a NIR spectrum thereby unveiling its inherent complexity should be particularly noted. A good example is provided in Figure 11, in which the individual contributions from overtones and combination bands are presented in common scale with the observable NIR spectrum. This example demonstrates well the extent of band overlapping in a NIR spectrum because of its convoluted nature, and explains the difficulty for its interpretation using conventional methods of spectral analysis. The overviewed accomplishments yield useful information for direct analysis of NIR spectra of biological systems [89] and are critical for increasing the interpretability of such spectra.

#### 3.7.2. Nucleobases

Nucleic acids are typically minor constituents, and hence a clear appearance of their bands in NIR spectra is unlikely. Detailed assignments of NIR bands of nucleobases (nucleic acid bases) are essential for the possibility to unveil the characteristic wavenumbers as markers for nucleic acids present in the sample [94]. It was possible to construct a frequency correlation table for NIR spectra of purines and pyrimidines. The study found that in-plane deformation bands are far more populated in NIR spectra of nucleobases than out-of-plane ones, and the significance of ring modes is relatively minor. Such a trend is different to that observed in the IR (mid-IR) region [95]. Moreover, it was concluded that local, short-ranged chemical neighborhood of nucleobases molecules influence their NIR spectra relatively more than the IR spectra. Consequently, it seems that NIR spectra are a more sensitive probe of the nucleobase pairing than IR spectra. Dissection of NIR spectra of nucleobases in the crystalline state yields important information on the spectral patterns resulting from the interaction with the chemical neighborhood. This factor is of great interest for nucleic acids as well.

#### 3.7.3. Active Pharmaceutical Ingredients (APIs) in Medicinal Plants

**Figure 12:**
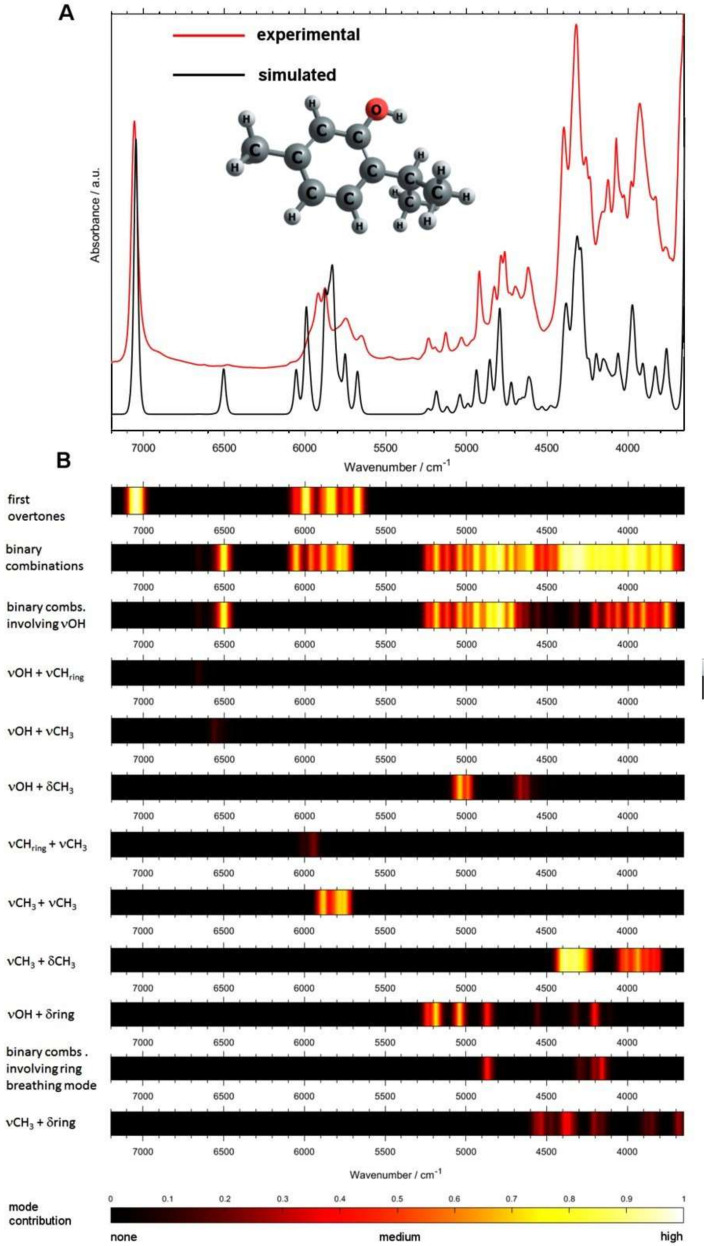
The analysis of mode contribution for the NIR spectrum of thymol (solution; 100 mg mL^−1^ CCl_4_) based on the simulated data. (**A**) Experimental and simulated outlines. (**B**) Contributions of selected modes as described on the figure. Reproduced with permission from Ref. [96].

Bio-active constituents of medicinal plants and natural products have been examined with help from quantum chemical calculation of NIR spectra as well. Examples include thymol [96], rosmarinic acid [75], caffeine and L-theanine [97]. In particular, the studies of thymol supported by spectra simulation opened a new trend-setting research direction, as they yielded fundamental findings about the relationship between the specific vibrational modes and the features of PLS regression coefficients vector [96]. Band assignments unveiled for thymol are provided in Figure 12 as an informative example. Thymol is a terpene giving the traditional plant medicine *Thymi herba* its therapeutic properties. The drug features in the European Pharmacopeia. Thymol features several curative values, e.g., anti-inflammatory, immunomodulatory, antiseptic, antifungal, antibacterial, antispasmodic, and anti-oxidant properties. In addition to its phytopharmaceutical significance, the thymol molecule forms an interesting object for spectroscopic investigation. The side groups attached to an aromatic ring have characteristic influence on the NIR spectrum; that investigation brought important and universal information as phenolic moieties are common for a large number of chemicals present in plants and other biological systems. The OH group gives thymol the capacity to strongly interact with its chemical surroundings through forming a hydrogen-bond network. It is well-known that the intense bands from OH group are important for shaping NIR spectra.

The investigation of NIR spectra of thymol by Beć et al. [96] included dependence of the sample state (solid, melted, soluted) and concentration (for soluted sample). The unveiled patterns of spectral changes evidenced existence of NIR bands with varying levels of sensitivity to the intermolecular interactions. This can be interpreted as the general sensitivity to the matrix effects. Two NIR wavenumber regions (A: 6000-5600 cm^−1^ and B: 4490-4000 cm^−1^) were identified with bands not overly affected by the sample state. Further, interpretation of the regression features in the quantification of thymol content in *Thymi herba* was performed. The analysis of PLS regression coefficients vector unveiled the most influential absorbances to be located in these two NIR spectral regions. Quantum calculation of NIR spectra provide detailed origin of the absorption bands of thymol (Figure 12). The contributions from vibrational transitions to NIR spectrum of thymol were projected giving complete overview, helpful in analysis of the complex absorption pattern characteristic for NIR spectra (Figure 12). This enabled comprehensive dissection of the matrix effects. The region A was found to be mostly populated by the combinations and overtones of C-H and CH_3_ stretching modes. The absorption in the region B is primarily due to combinations of CH_3_ stretching and deformation modes and secondary influences from the combinations involving ring deformations. It was found that the abundant bands due to OH vibrations were not recognized as the influential factors in the PLS regression. This finding contradicts one’s anticipation as the most significant and characteristic OH vibrations that are the most sensitive to the chemical surrounding do not correlate well with the thymol content in the sample. These conclusions shed new light on the matrix effects and demonstrate the potential stemming from chemometrics used in hyphenation with quantum chemical spectra calculations [96].

#### 3.7.4. Carbohydrates in Aqueous Environment

**Figure 13:**
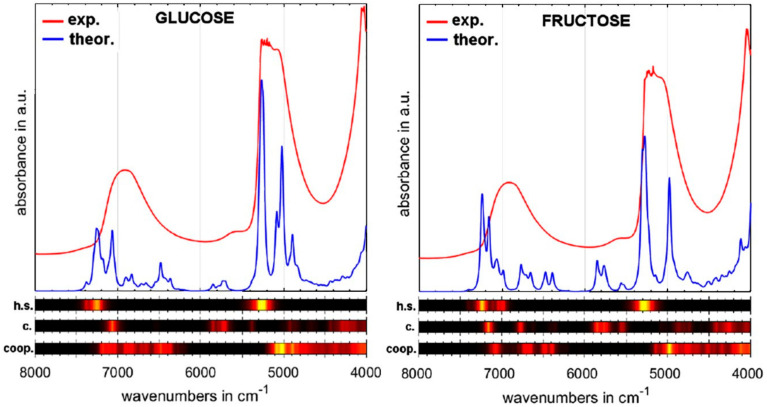
Simulated spectra of the carbohydrate-hydration shell system on the example of glucose and fructose. The color bars represent the relative spectral contributions (color scale: yellow—high; black—none) from: pure vibrations of hydration shell (h.s.); pure vibrations of carbohydrate (c.); cooperative vibrations of carbohydrate and hydration shell (coop). Reproduced in compliance with CC BY 4.0 license from Ref. [104].

In the context of the reviewed topic, carbohydrates in aqueous environment are essential molecules for several reasons. Analysis of glucose in body fluids and in tissue is one of the most frequent applications of NIR spectroscopy in biomedical science and in clinical practice (e.g., Section 3.1.1). Further, these molecules are the center of interest of analytical applications of NIR spectroscopy in the agriculture and food sector [98,99,100]. Finally, the physiochemical properties of carbohydrates and their interaction with solvent are important [101,102,103]. Therefore, they attract considerable attention in vibrational biospectroscopy [7]. Recently, quantum chemical calculations of NIR spectra of six carbohydrates (glucose, fructose, mannose, ribose, xylose and sorbitol) were used to support the examination of these carbohydrates in aqueous solution at different concentration levels (5 mg/L, ~0.03 mmol/dm^3^; and 20 mg/L, ~0.1 mmol/dm^3^) [104]. The simulation unveiled that vibrational coupling between a carbohydrate and its hydration shell is highly wavenumber-selective. It leads to an enhancement of the qualitative information contained in the specific spectral regions, and improves the classification accuracy by PLS-DA method at very low concentration levels [104]. This study brings new insight to the effects on vibrational spectrum of coupling to hydration shells and cooperative vibrations of a hydrated molecule and water molecules in its hydration shell. Interaction with hydration shell is an intensively studied topic by vibrational spectroscopy [105]. An example of a calculated NIR spectrum of glucose and fructose is presented in Figure 13; the simulated spectra of mannose, ribose, xylose and sorbitol are available in Ref. [104].

### 3.8. Introduction to Functional NIR Spectroscopy (fNIRS)

Functional NIR spectroscopy (fNIRS) is an optical topography (i.e., imaging) technique used for monitoring brain function in clinical applications. It is an area of medicine, in which NIR spectroscopy exemplified a unique potential and has been extensively adopted for research and diagnosis. One can find a remarkably rich scientific and medical literature devoted to fNIRS, which well reflects the vigorous discussion over its development and applications. For this reason, it falls beyond the capacity of this work to discuss in detail the applications of fNIRS. Instead, the discussion will focus on introducing the fundamental principles of this technique, while interested reader will be pointed to the most relevant exhaustive literature covering key discussed aspects of fNIRS.

**Figure 14:**
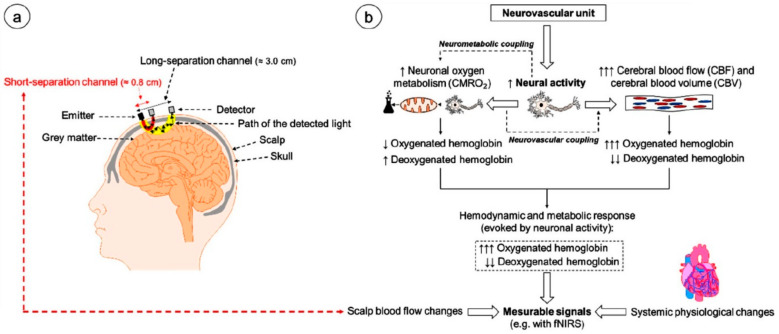
(**a**) Schematic illustration of the neurovascular unit and the changes in cerebral hemodynamics and oxygenation induced by neural activity. (**b**) Exemplary illustration of a possible NIRS montage on the human head and the assumed banana-shaped course of detected light of “short-separation channels” and of “long-separation channels”. fNIRS, functional near-infrared spectroscopy; CMRO2, cerebral metabolic rate of oxygen; increase; decrease. Reproduced in compliance with CC BY 4.0 license from Ref. [113].

Previous sections mentioned the typical high permeability of organic materials to NIR radiation. The adoption of NIR spectroscopy for non-invasive sensing of brain function is possible as the transmissivity through biological tissue (so-called “Near-infrared window in biological tissue”) is high enough to enable percutaneous measurement of cerebral cortex located beneath the skull [106]. Brain neuro-activity (i.e., firing of nerve cells) influences active energy metabolism in the cerebral cortex with an increase in blood volume and blood flow to supply glucose and oxygen to brain as the secondary effects. Hence, through neuro-vascular coupling the neural activity is correlated with the blood volume change, and the latter parameter is an accurate index of the brain activity (Figure 14). The local changes in cerebral blood volume due to neural activity can be accurately monitored by NIR spectroscopy. This is achieved through measuring NIR signal of hemoglobin and its oxidation state as the sensitive marker. The absorption differs depending on the oxygenation level of hemoglobin. The two oxidation states of hemoglobin (Oxy- and Deoxy-Hb) differ in their NIR absorption spectrum (Figure 14). The hemoglobin concentration level is determined through the modified Beer-Lambert law. The development of a quantitative approach to Hb concentration was described by Yamashita et al. [107]. Besides NIRS-derived oxygenation indexes, NIR spectroscopy in conjunction with the tracer indocyanine green dye (ICG) has been used on assessing cerebral cortex perfusion in healthy individuals, patients, and critically ill population [108,109,110,111,112]. An exhaustive discussion of the basic principles and methods used in fNIRS is available in a recent review article by Herold et al. [113].

Nowadays, the instrumentation and methodology of fNIRS improved (e.g., in terms of portability, spatial resolution, time-to-result) and is highly suitable for imaging of brain activity in real-time with minimal inconvenience for patients. Autonomous fNIRS sensors configured as wireless wearables enable continuous monitoring of patient’s brain activity throughout his normal daily life. The neural activity in cerebral cortex is associated with high level functions such as motion, sensation, perception, speaking, etc. This makes it suitable for fNIRS to be used as a substantial aid in diagnosing even complex problems, e.g., in psychiatry [114]. Moreover, fNIRS provides valuable research data indispensable for advancing neuroscience and our basic understanding of the higher functions of brain and improving rehabilitation therapies [115].

It should be noted that, there are other techniques established in medical practice for non-invasive assessment of brain activity, e.g., functional magnetic resonance imaging (fMRI), positron emission tomography (PET), magnetoencephalography (MEG), electroencephalography (EEG), each with specific advantages and disadvantages. Brief comparison of the characteristics of fNIRS opposed to the competitive technique is provided beneath. The prime advantages of fNIRS are simplicity and safety of the instrumentation. Unlike MEG or PET, no high-energy photon radiation is used, meaning that no radiation exposure risk exists and an ordinary non-shielded room is suitable. For the same reason, repeated measurements can be performed on relatively more vulnerable subjects, e.g., infants or elders. Safety-related aspects of fNIRS are exhaustively discussed in the literature [116,117]. Unlike fMRI, fNIRS is convenient for patient, as the diagnosis can be performed with the subject holding a natural posture (sitting, standing or lying). The sensor require minimum wiring or can be wireless, hence the subject can be diagnosed under motion and activity. Finally, fNIRS is vastly superior to all other functional imaging modalities in its cost-effectiveness and portability. While the cost factor might be negligible in the developed world, it should be emphasized that this particular benefit is critical to wide-spread modern medical imaging diagnostic tool in the developing countries such as the African continent [118,119].

For further information of fNIRS, an interested reader may consider review articles describing the history of this discipline by Ferrari and Quaresima [120] or by Boas et al. [121]. Systematic discussion of the methodology, in a tutorial-resembling form is available from Herold et al. [113]. A solid review of the general principles and applications of fNIRS is available in a form of review article by Irani et al. [122]. Comprehensive discussion of the recent advances and applications of fNIRS throughout different areas of medicine can be found in review articles, e.g., by Yang et al. (stroke diagnosis and rehabilitation) [123], Mihara and Miyai (neurorehabilitation) [115], or Ernst et al. (applications in psychiatry) [114]. Novel concepts of sensor miniaturization and autonomy in the form of wireless wearables were described in detail by Pinti et al. [124], current state of knowledge in the area of preprocessing of fNIRS imaging data were dissected by Pinti et al. [125], while prospects for future advances of fNIRS in the whole were summarized by Quaresima and Ferrari [126].

### 3.9. Selected Other Applications

For sake of completeness, a brief note about certain other fields of application of NIR spectroscopy in which samples of biological origin are typically examined. This technique has become well recognized in ecology and environmental studies [127]. NIR spectroscopy is easily applicable and delivers highly useful information on a remarkably wide variety of sample types in the scope of ecology. Not only direct examination of living specimens of flora and fauna is possible by NIR spectroscopy, but also various properties of habitat, environment, feed, foraging, or feces. Further, spectroscopic analysis is in its essence chemical reagent-free, and as such, spectroscopy itself is environmental friendly. A focused review of the role of NIR spectroscopy in modern research in the field of ecology and environment is available in recent literature [127].

**Figure 15:**
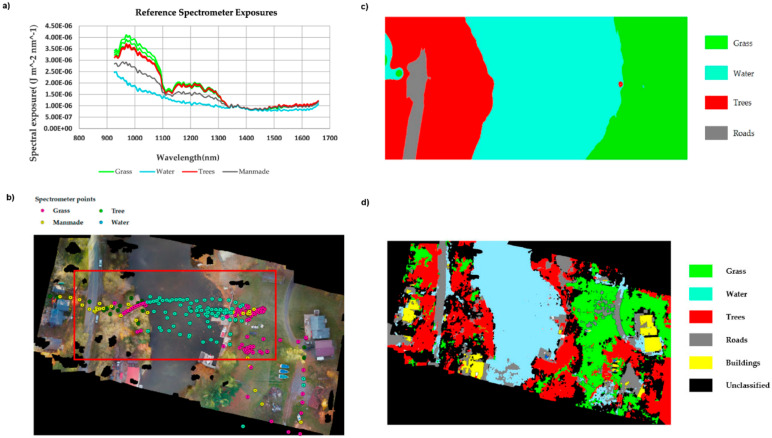
Discrimination between terrain types and different types of vegetation based on a cost-effective UAV NIR spectroscopy. (**a**) Reference NIR spectral signatures associated to the elements of terrain and vegetation; (**b**) the reference Vis (RGB) multispectral image; (**c**,**d**) classification maps derived from NIR spectral analysis using different methods of classification for image generation. Adopted in agreement with CC BY 4.0 license, from Ref. [128].

Attention should be given to NIR spectrometers and imaging instrumentation mounted on unmanned aerial vehicles (UAVs). Sensor miniaturization, new low-power technology, and progress in spectral data processing enabled successful use of spectroscopy on airborne drones. This forms a decisive advancement for agriculture and environmental applications of NIR spectroscopy, where high-throughput, remote sensing of large Earth surface areas becomes feasible. In such applications, the NIR spectral footprint of plants is often in the center of attention. Agricultural applications typically put emphasis on inspecting the condition and properties of plants. However, efficient methods for basic discrimination and classification of vegetation are under continuous development enabling cost-effective airborne NIR spectroscopy [128]. The importance of the techniques of spectral data analysis and image generation should be highlighted; the impact on the final information contained in the spectral image is demonstrated by the examples provided in Figure 15. Current developments in airborne spectroscopy, including NIR, were reviewed recently [129].

NIR spectroscopy is a powerful tool in exploration of the structure and properties of water. Significance of water as a biological environment, and its properties as well as interactions with other molecules, biomolecules in particular, are essential for understanding of biological processes. NIR spectroscopy substantially contributed to shedding light on the structure of water, e.g., early advances by Segtnan et al. [130]. Water absorbs relatively weaker in NIR region than IR region, and simultaneous observation of the characteristic bands of the solvent and soluted molecules is possible [131]. An exhaustive review of the research advancements into the structure and properties of water by NIR spectroscopy was published recently [132].

## 4. Summary and Future Prospects

NIR spectroscopy offers immense potential in various bio-applications with its primary advantages; rapid analysis, wide-applicability to variety of samples, capability of examining moist samples, flexible instrumentation including miniaturized sensors enabling portability. On the other hand, in certain fields it is still a developing discipline, with room for improvement as compared with the competing techniques. The area of bioanalytical research and medical diagnosis should be mentioned here, where it still faces strong competition from IR and Raman spectroscopy. However, recent literature indicates that NIR spectroscopy steadily conquers new fields of application. Current progress includes improvements in the interpretability of NIR spectra, shortening the distance to IR or Raman techniques typically valued for their superior chemical specificity. This becomes particularly significant in the case of chemically complex samples of biological origin. Novel handheld NIR spectrometers are indispensable in on-site examination of biosamples, e.g., medicinal plants with aim to optimize the cultivation conditions and ensure highest quality of natural drugs. Progress on the instrumentation recently enabled engineering remote airborne NIR sensors deployable on UAVs for environmental monitoring. This is supplemented by advances in data-analytical methods, also stimulated by other fields. It is expected, that in the forthcoming decade NIR spectroscopy will further extend its usefulness across the field of bio-applications, with the continuation of current lanes of research and the appearance of entirely new ones.

## Figures and Tables

**Table 1:** Approximate positions of NIR bands that are meaningful for samples of biological origin (excluding water). In brackets provided are the exemplary chemical compounds for which the transitions are specific. The assignments adopted from literature [9,11].

Wavenumber in cm^−1^	Wavelength in nm	Vibrational Mode Assignment and the Associated Most Characteristic Compounds ^a)^
8250	1210	3 C–H str. (C-H rich compounds, e.g., carbohydrates, lipids)
7375–7150	1355–1400	2 C–H str. + C–H def. (carbohydrates, lipids)
6980	1435	2 N–H str. (proteins)
6750	1480	2 O–H str. (carbohydrates, alcohols, polyphenols)
6660	1500	2 N–H str. (proteins)
6500	1540	2 O–H str. (carbohydrates, alcohols, polyphenols upon matrix effects, e.g., hydrogen bonded OH groups)
6400	1565	2 N–H str. (proteins)
6200–5800	1610–1725	2 C–H str. (carbohydrates, lipids)
5625	1780	2 C–H str. (C-H rich compounds, e.g., carbohydrates, lipids)
5500	1820	O–H str. + 2 C–O str. (carbohydrates)
5120	1955	3 C–O str. (carbohydrates)
4880	2050	N–H sym. str. + amide II (proteins)
4825	2075	O–H str. + O–H def. (alcohols, polyphenols)
4645	2155	Amide I + amide III (proteins)
4440	2255	O–H str. + O–H def. (carbohydrates, alcohols, polyphenols)
4360	2295	N–H str. + CO str. (proteins)

^a)^ Numbers two and three denote the first and second overtones, respectively; plus sign (+) denotes combination bands.

**Table 2:** Basic characteristics of NIR spectroscopy in comparison with competing techniques.

	NIR	IR	Raman
**Spectral region** **wavelength [nm]** **wavenumbers [cm^−1^]**	1000–2500 10,000–4000	2500–25,000 4000–400	2500–200,000 4000–50
**excitation mechanism**	absorption	absorption	inelastic photon scattering
**relative complexity of instrumentation**	low	medium	high
**selection rule (chemical sensitivity)**	change in dipole moment (polar moieties, enhanced signal of X–H groups, e.g., O–H, N–H, C–H)	change in dipole moment (polar moieties)	change in polarizability (non-polar symmetrical bonds, e.g., C–C, skeletal vibrations)
**sampling (i.e., spectra acquisition modes)**	transmission; diffuse reflection; transflection	transmission; diffuse reflection (only after sample preparation); transflection; attenuated total reflectance (ATR)	reflection (scattering)
**remarks about sample preparation**	no/minimal sample preparation needed; moderate suitability of water as solvent or glass as container/optics	optimal sample thickness (in transmission mode); sample dilution (e.g., KBr pellet) for diffuse reflectance mode; optimal/stable sample-IRE contact surface (in ATR mode)	suitability of water as solvent or glass as container/optics
**chemical specificity**	low to moderate	high	high
**major issues and challenges**	low sensitivity; overlapping contributions in the spectra; difficult spectral interpretation;	limited suitability of moist samples; unsuitability of glass optics and materials (absorption of glass); interfering signal from atmospheric H_2_O and CO_2_	Raman signal obscured by autofluorescence (stronger for excitation lasers with shorter emission wavelengths); laser heating, danger of destruction of molecular structure, e.g., proteins; or sample thermal decomposition (particularly of dried material)

Abbreviations: IRE - internal reflection element.

**Table 3:** Performance parameters of prediction of blood constituents by partial least squares (PLS) regression models developed for IR and NIR spectra of a 5-component model mixture in artificial dialysate solutions. Reproduced in agreement with CC BY 4.0 license, from Ref. [33].

Model			Factor	*R* ^2^	RMSECV in mg/dL	RMSEP in mg/dL	LOD_min_ in mg/dL	LOD_max_ in mg/dL	LOQ_min_ in mg/dL	LOQ_max_ in mg/dL
Urea	CV	NIR	4	0.97	12	-	10	24	29	72
		IR	4	0.99	7.9	-	10	18	31	55
	TV	NIR	4	0.98	-	19	-	-	-	-
		IR	5	0.99	-	6.6	-	-	-	-
Glucose	CV	NIR	4	0.89	37	-	36	73	108	218
		IR	3	0.96	22	-	47	142	140	428
	TV	NIR	4	0.86	-	54	-	-	-	-
		IR	2	0.99	-	11	-	-	-	-
Lactate	CV	NIR	-	-	-	-	-	-	-	-
		IR	5	0.95	8.2	-	28	90	84	271
	TV	NIR	-	-	-	-	-	-	-	-
		IR	8	0.99	3.0	-	-	-	-	-
Phosphate	CV	NIR	-	-	-	-	-	-	-	-
		IR	8	0.99	1.1	-	1.0	2.6	3.0	7.9
	TV	NIR	-	-	-	-	-	-	-	-
		IR	8	0.95	-	2.0	-	-	-	-
Creatinine	CV	NIR	-	-	-	-	-	-	-	-
		IR	5	0.98	1.8	-	2.6	4.5	7.9	13
	TV	NIR	-	-	-	-	-	-	-	-
		IR	4	0.96	-	2.1	-	-	-	-

Abbreviations: RMSECV—root mean square error of cross-validation; RMSEP—root mean square error of prediction; LOD—limit of detection; LOQ—limit of quantification; CV—cross validation; TV—test-set validation; *R*^2^ —coefficient of determination.

**Table 4:** Absorption regions that are meaningful for characterizing cancer tissue by NIR spectroscopy. For sake of completeness, listed are wavelength regions extended to Vis region.

Wavenumber Region [cm^−1^]	Wavelength Region [nm]	Parameters Measured	Ref.
15,873–11,111	630–900	changes in hemoglobin oxygenation; quantification of total hemoglobin; tissue oxygen saturation	[52]
10341, 10204, 8665, 8368, 7133, 6925, 5297, 5144	967, 980, 1154, 1195, 1402, 1444, 1888, 1944	water	[72]
6798, 5233	1471, 1911	DNA	[72]
4866, 4604, 4261	2055, 2172, 2347	proteins	[72]
9000–7905 6000–5500	1111–1265 1666–1818	lipids	[72]
15,385–25,000	650–400	tissue scattering profile	[73]

**Table 5:** Performance parameters of PLS regression models for the prediction of rosmarinic acid content C_RA_ in *Rosmarinus officinalis*, folium. Reproduced with permission from Royal Society of Chemistry, Ref. [75].

Spectrometer		NIRFlex N-500		microPHAZIR		MicroNIR 2200	
samples		60		60		60	
outliers		6		8		4	
*C*_RA_ (*w*/*w*) range/%		1.138–2.425		1.138–2.425		1.138–2.425	
validation method		CV	TSV	CV	TSV	CV	TSV
*R* ^2^		0.91	0.91	0.73	0.73	0.84	0.85
SECV/%	SEP/%	0.072	0.069	0.12	0.11	0.091	0.11
SECV/SEC	SEP/SEC	1.46	1.43	1.28	1.24	1.55	2.09
factors		8	8	5	5	11	12
RPD		3.27	3.41	1.88	2.06	2.46	2.14

Abbreviations: CV—cross validations; TSV—test-set validation; SECV—standard error of cross validation; SEP—standard errors of prediction; SEC—standard error of calibration; *C*_RA_ —rosmarinic acid content (*w*/*w*).
